# Assessment of the correlation of commonly used laboratory tests with clinical activity, renal involvement and treatment of systemic small-vessel vasculitis with the presence of ANCA antibodies

**DOI:** 10.1186/s12882-021-02495-8

**Published:** 2021-08-26

**Authors:** Magdalena Mosakowska, Dorota Brodowska Kania, Katarzyna Szamotulska, Aleksandra Rymarz, Stanisław Niemczyk

**Affiliations:** 1 Department of Internal Diseases, Nephrology and Dialysis, Military Institute of Medicine, 128 Szaserów Street, 04-141 Warsaw, Poland; 2 Department of Epidemiology and Biostatistics, Institute of Mother and Child, 17a, Kasprzaka Street, 01-211 Warsaw, Poland

**Keywords:** ANCA, vasculitis, microhematuria, CRP, ESR, procalcitonin, fibrinogen, d-dimer, complement system

## Abstract

**Background:**

The aim of the study was to assess the correlation of commonly used laboratory tests with clinical activity, degree of kidney involvement and treatment of systemic small-vessel vasculitis with the presence of ANCA antibodies.

**Methods:**

The study included 28 patients with active AAV (BVAS ≥ 3). The following tests were performed: MPO-ANCA, PR3-ANCA, peripheral blood count, ESR, CRP, procalcitonin, creatinine, GFR, urea, albumin, fibrinogen, d-dimer, components of the C3 and C4 complement systems, urinalysis with sediment evaluation and diurnal proteinuria. The assessments were conducted twice: at study entry (A0) and after 6 months (A6) (BVAS = 0).

**Results:**

At the time of inclusion in the study, the mean creatinine concentration was 3.39 mg/dl (GFR 33.17 ml/min/1.73 m²), after achieving remission in 11 patients (39.3 %) GFR remained below 30 ml/min/1.73 m², 4 patients (14.3 %) continued renal replacement therapy, and 3 patients (10.7 %) with advanced renal failure died. Microscopic hematuria occurred in 80.9 % of the studied population, withdrew in most patients, strongly correlated with renal involvement *p* < 0.001 and was not related to disease severity *p* = 0.147. CRP, ESR, fibrinogen, d-dimer, albumin and hemoglobin in the peripheral blood showed a strong correlation with the clinical activity of AAV and well identified severe patients. High procalcitonin concentrations correlated with a severe form of the disease, pulmonary involvement with respiratory failure and alveolar hemorrhage (mean 3.41 ng/ml, median 0.91 ng/ml, SD 7.62, *p* = 0.000), and were associated with the occurrence of infectious complications and the need to administer antibiotic therapy. ANCA antibodies were useful in the evaluation of patients with AAV, the amount of antibodies did not correlate with the severity of vasculitis (*p* = 0.685) and the results in many patients did not match the expected assumptions.

**Conclusions:**

CRP, ESR, fibrinogen, d-dimers, albumin and hemoglobin in the peripheral blood correlate well with the activity of vasculitis and identify severe patients. The resolution of microscopic hematuria suggests remission of the disease in the renal area. Procalcitonin may be slightly increased in patients with active AAV without infection, high concentrations are strongly associated with infectious complications. ANCA antibodies should always be interpreted in the context of the observed clinical symptoms.

## Introduction

ANCA associated vasculitis (AAV) is associated with necrotic inflammation in the wall of small arteries, veins and capillaries [1, 2]. The consequence is ischemia, followed by impairment of the organ functions. The classification of AAV includes granulomatosis with polyangiitis (GPA), microscopic polyangiitis (MPA), eosinophilic granulomatosis with polyangiitis (EGPA) and renal limited-AAV (RLV). AAV is a rare disease, the annual incidence is estimated at 5.0-10.65/million. The five-year survival rate for GPA is 74–91 % and for MPA 45–76 % [3, 4]. ANCA antibodies are detected in the serum in approximately 90 % of patients, and in 10 % they are absent despite the typical clinical course. The sensitivity of the detected PR3-ANCA and MPO-ANCA amounts to 85.5 %, and the specificity is as high as 98.6 % [3, 4]. Controversies concern the usefulness of testing for antibodies in monitoring vasculitis activity [5]. Patient evaluation is based on a multivariate analysis and requires extensive clinical experience. In everyday clinical practice, we observe an incomplete correlation of test results with the condition of a patient and the intensity of involvement of various organs. This applies, in particular, to the markers of inflammation, i.e. peripheral blood leukocytosis, ESR, CRP, or the amount of ANCA antibodies. Additionally, the clinical picture includes symptoms resulting from permanent organ damage that occurred during the disease, adverse events of drugs and infectious complications. The absence of markers unequivocally correlating with AAV activity and differentiating between other clinical conditions makes diagnostic and treatment difficult. The objective of the study was to assess the correlation of commonly used laboratory tests with clinical activity, degree of renal involvement and treatment of systemic small-vessel vasculitis with the presence of ANCA antibodies.

## Materials and methods

The study included 28 patients with active AAV. In 16 patients the disease was newly diagnosed, and in 12 patients another exacerbation was observed. The control group included 27 patients without diagnosed autoimmune disease, matched in terms of age, sex and stage of chronic kidney disease to patients from the study group. The criteria for inclusion in the study were: informed consent of the patient, age over 18 years, diagnosed small-vessel vasculitis with the presence of ANCA antibodies, meeting the ACR 1990 criteria and in accordance with the CHCC 2012 nomenclature. The exclusion criteria were the lack of consent to participate in the study, inflammation of small vessels without the presence of ANCA antibodies at the diagnosis of the disease, or lack of cooperation with the patient. Patients without the presence of ANCA antibodies were excluded from the study due to possible doubts in AAV diagnosis. Additionally, an important aim of the study was the analysis of relationship between ANCA levels and activity and severity of vasculitis and its changes after induction therapy. We obtained written informed consent from all participants. All methods were performed in accordance with the relevant guidelines and regulations.

 The study protocol was approved by the Bioethics Committee of the Military Institute of Medicine in Warsaw on June 18, 2014 (No. 26/WIM/2014). The study was funded by a Grant for a Young Researcher of the Military Institute of Medicine.

A detailed analysis of clinical data was performed, disease activity and remission were verified using the BVAS/WG and BVAS v3 scales.

The following laboratory tests were performed: MPO-ANCA (positive result > 5.0 IU/ml), PR3-ANCA (positive result > 3.0 IU/ml), peripheral blood count, ESR, CRP, procalcitonin (PCT), creatinine, GFR, urea, albumin, fibrinogen, d-dimer, components of the C3 and C4 complement systems, urinalysis with sediment evaluation and diurnal proteinuria. Patients from the study group were assessed twice: at study entry (A0) and after 6 months (A6) in the early remission stage (BVAS = 0). Patients from the control group were matched in terms of age, gender, stage of chronic kidney disease, GFR, and the need for renal replacement therapy with patients with AAV. The control group did not include patients with systemic or autoimmune diseases or the diagnosis of glomerulonephritis.

Disease severity was defined as follows: Localized form: one organ involved, most often the upper respiratory tract, no general symptoms, creatinine concentration < 120 mol / l (< 1.34 mg / dl). Early systemic: any organ may be involved without its failure, general symptoms may occur, no kidney involvement, creatinine concentration < 120 mol / l (< 1.34 mg / dl).Generalized: life-threatening symptoms or kidney involvement, creatinine < 500mmol / l (< 5.6 mg / dl), present general symptoms.Severe: organ failure, alveolar bleeding, respiratory failure, renal failure, creatinine > 500mmol / l (> 5.6 mg / dl), present general symptoms.

During the study, 2 patients from the study group died in the course of septic shock in the early stages of the disease, shortly after the introduction of immunosuppression, 1 person died 6 weeks after the inclusion due to severe Clostridium difficile infection and 2 individuals were disqualified as a result of non-cooperation. The remaining 23 patients were included in the comparative analysis at baseline and after 6 months of treatment.

Basic descriptive statistics such as arithmetic mean, median, standard deviation, minimum and maximum values were employed. The hypotheses regarding comparisons between the two groups were verified using the non-parametric Mann-Whitney test and the Wilcoxon test for small study groups, respectively. The verification of hypotheses concerning comparisons between more than two groups was performed using the non-parametric Kruskal-Wallis test. Trend analyzes were done with the use of the Jonckheere-Terpstra test. In the case of categorized variables, the Fisher test was employed. The significance level was set at 0.05.

## Results

In the study population with active AAV, 67.9 % were women. The average age was 58.3 years. GPA was diagnosed in 16 patients (57 %) and MPA in 12 patients (43 %). The clinical activity of the disease was evaluated according to the BVAS/WG scale at 8.46 pts (min. 3 pts, max. 16 pts) and according to the BVAS v3 scale at 19.14 pts (min. 7 pts, max. 31 pts). The localized form was diagnosed in 1 person, the early systemic form in 5 people and the dominant form was generalized and severe vasculitis (22 patients; 78.6 %) [Table 1]. The lungs were involved in 24 individuals (86 %), and the upper respiratory tract in 15 (54 %). HRCT was dominated by ground-glass opacities and parenchymal infiltrates. Hemoptysis occurred in 9 patients while respiratory failure requiring mechanical ventilation or assisted respiration developed in 6 individuals.

**Table 1:** Characteristics of the studied population

	AAV group (A) (*N* = 28)	Control group (C) (*N* = 27)
**Age**, mean ± SD, years	58.32 ± 15.14	60.3 ± 17.14
**Sex**, females/males (%)	19 (67.9 %) / 9 (32.1 %)	18 (66.6 %) / 9 (33.3 %)
**ANCA** PR3/MPO	17 (71 %) / 11 (29 %)	-
**BVAS v3, mean** ± SD	19.14 ± 7.14	-
**BVAS/WG, mean** ± SD	8.46 ± 3.66	-
**Organ activity** skin joints upper respiratory tract lung kidneys/HD nervous system	7 (25 %) 24 (86 %) 15 (54 %) 24 (86 %) 21 (75 %) / 12(43 %) 7 (25 %)	-
**Clinical form** localized and early systemic generalized severe	6 (21.4 %) 10 (35.7 %) 12 (42.8 %)	-
**Remission induction** glucocorticoids plasmapheresis cyclophosphamide rituximab other	28 ( 100 %) 7 (25 %) 18( 64 %) 6 ( 21 %) 4 (14 %)	-

In 21 patients (75 %), the kidneys were involved The creatinine concentration increased by more than 30 % and/or the presence of microscopic hematuria (≥ 10 erythrocytes in the field of view) was confirmed. 9 patients (32 %) started renal replacement therapy, and 3 persons with subsequent AAV exacerbation (11 %) were treated with hemodialysis as a result of earlier disease activity. After 6 months, only 3 patients were able to complete renal replacement therapy, and the remaining 6 patients continued dialysis treatment. Within 6 months of follow-up, 3 patients (10.71 %) died due to infectious complications, in each case severe AAV was diagnosed. In the remission-inducing treatment, all patients received GCS, in 24 patients (85.71 %), oral prednisone administration was preceded by repeated infusions of methylprednisolone at 0.25-1.0 g/day for 3–5 days. 18 patients (64 %) received cyclophosphamide according to the protocol from the CYCLOPS study, rituximab at a dose of 375 mg/m² of body surface area in four weekly infusions was administered in 6 individuals (21 %), mycophenolate mofetil in 2 patients (7 %) and azathioprine (increase in chronic doses during the exacerbation of the disease) in one person (4 %). 7 patients (25 %) underwent therapeutic plasma exchange. After 6 months of immunosuppressive treatment, remission was confirmed (BVAS v3 = 0 pts, BVAS/WG = 0 pts). Organ complications resulting from the underlying disease and/or immunosuppressive treatment were evaluated according to the VDI scale (mean = 3.5 pts, min − 0 pts, max − 9 pts).

In the studied population, the mean initial concentration of creatinine was high and amounted to 3.39 mg/dl (mean GFR 33.17 ml/min/1.73 m²) and after 6 months of treatment it decreased only to 2.86 mg/dl (mean GFR 37.87 ml/min/1.73 m²). The difference was not statistically significant [Table 2].

**Table 2:** Laboratory test results - assessment of kidney involvement

	Initially active AAV (A0)	AAV remission(A6)	P *	Control group	P**
**Creatinine**(mg/dl)	2,7 (1,4;5,6)	2,1(1,3;3,8)	0.051	2,5(1,4;7,5)	0.427
**eGFR** (ml/min/1.73 m²)	18,0(8,0;51,0)	24,0(13,0;60,0)	0.054	22,0(6,0;59,0)	0.436
**Urea**(mg/dl)	80,0(44,0;162,0)	84,0(47,0;117,0)	0,123	94,0(43,0;125,0)	0,298
**Proteinuria** (g/24 h)	0,51(0,27;1,2)	0,47(0,13;0,93)	0.275	0,23(0,15;0,79)	0.425
**Hematuria**> 4	81.9 %	18 %	**< 0.001**	19 %	**< 0.001**

p* comparison of patients with active vasculitis and in remission, p** comparison of patients with active vasculitis and the control group

Only in 4 patients (14.3 %) the GFR value increased above 60 ml/min/1.73 m², in 11 (39.3 %) it remained below 30 ml/min/1.73 m², including 3 patients (10.7 %) had to continue renal replacement therapy, 3 people (10.7 %) with advanced renal failure died. The amount of daily proteinuria did not show a significant correlation with AAV activity (*p* = 0.275), and the percentage of patients in remission with daily protein excretion above 150 mg was as high as 62.5 % [Tables 2 and 3].

**Table 3:** Parameters related to AAV activity within the kidneys depending on the severity of the disease

Disease form	Creatinine (mg/dl)	GFR(ml/min/1.73 m²)	Urea(mg/dl)	Proteinuria (g/24 h)	Hematuria
localized and early systemic *n* = 6	0,9(0,775;3,15)	90(39,75;90,0)	40 (33,75;67,25)	0,154(0,04;0,62)	0.0(0,0;3)
generalized *n* = 10	2,4(1,62;3,1)	26,5(15,5;47,5)	77 (50,0;97,9)	0,88(0,4;1,98)	3 (2,5;3,0)
severe *n* = 12	5,35(4,67;6,37)	9,5(7,25;12,0)	174,5(88,5;198,5)	0,85(0,42;1,2)	3 (2,0;3,0)
P *	**0,002**	**0,002**	**0,002**	**0,047**	0,077
P**	**0,000**	**0,000**	**0,000**	0,064	0,147

Values are median (IQR)

P * in the Kruskal-Wallis test. P** in the Jonckheere-Terpstra test for the trend

**Fig. 1:**
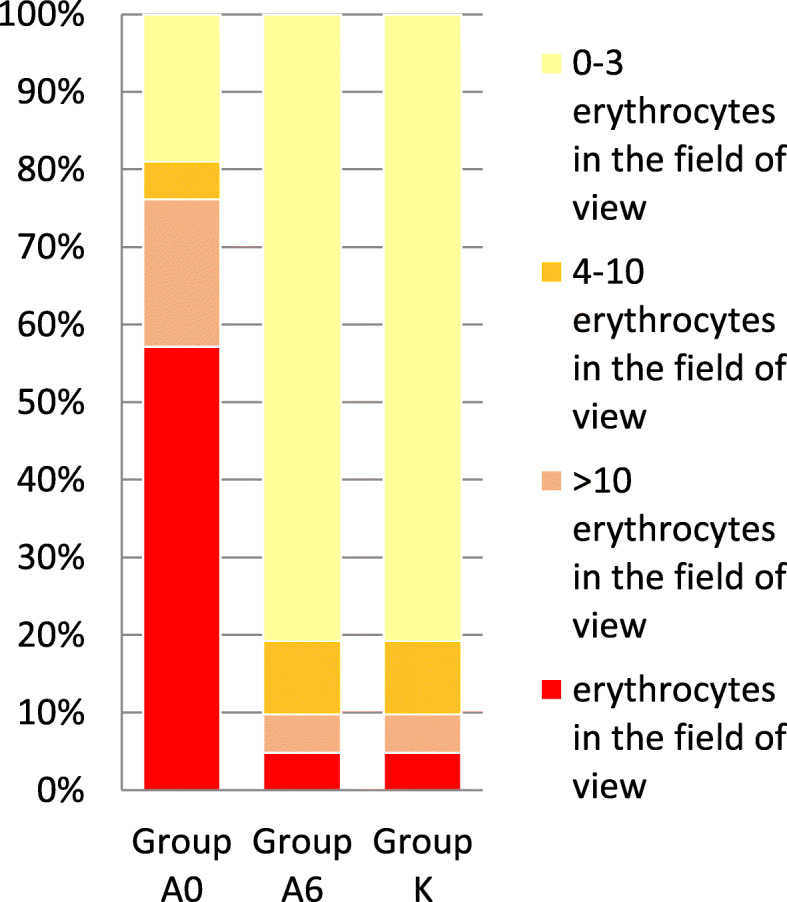
Severity of erythrocyturia in the sediment of a single urine sample.

The occurrence of microscopic hematuria was strongly related to AAV activity in the kidneys. In 57.1 % of patients, hematuria covering the field of view was observed, in another 23.8 % exceeded the value recognized as normal. After achieving remission, it withdrew in almost all patients, and the difference was statistically significant (*p* < 0.001) [Fig. 1].

At the time of AAV diagnosis, peripheral blood leukocytosis was significantly elevated compared to the control group (*p* < 0.001), the degree of leukocytosis increase did not correlate with the severity of the disease, and it did not decrease significantly in the follow-up, most likely due to the effect of GCS treatment (*p* = 0.249 ) [Tables 4 and 5]. Inflammation parameters of CRP and ESR showed a strong association with, both, activity and severity of AAV (*p* < 0.001). In the majority of patients, foci of infection were excluded, and peripheral blood and urine cultures were sterile. In the most severe patients, the exclusion of infection was very difficult, and it was necessary to administer broad-spectrum antibiotics. In order to differentiate high CRP values, procalcitonin was determined. The mean PCT concentration in patients with active AAV was 0.6 ng/ml, median 0.17 ng/ml and was significantly higher compared to the remission period of the disease (mean 0.19 ng/ml, median 0.08 ng/ml, *p* < 0.001 ). In 5 patients (22 %), PCT concentrations were: 0.98 ng/ml, 0.85ng/ml, 0.7 ng/ml, 1.01 ng/ml, 0.62 ng/ml respectively and were in the so-called a gray zone of 0.5 to 2.0 ng/ml despite no active infection. 2 female patients (9 %) with PCT of 4.17 ng/ml and 3.52 ng/ml had a severe form of vasculitis with pulmonary involvement, respiratory failure and alveolar hemorrhage, requiring broad-spectrum antibiotic therapy. 2 women who died in the course of septic shock (PCT: 27.28 ng/ml, 1.22 ng/ml) were excluded from the comparative analysis. In four cases with procalcitonin level above 1 ng/ml concomitant severe infections requiring antibiotic treatment were observed. In all cases with PCT lower than 1 ng/ml it’s elevation was associated with vasculitis activity. Procalcitonin concentration levels were particularly high in patients with severe AAV [Tables 4 and 5]. The concentration of fibrinogen in active AAV was significantly increased in 16 patients (69.5 %) and showed a strong relationship with the severity of the disease (*p* = 0.011). The concentration of platelets in the peripheral blood, although in most patients within the reference values, was statistically significantly higher in active AAV than in remission (289 thous./ul vs. 234 thous./ul; *p* = 0.024). However, it did not differ significantly from the results in the control group (*p* = 0.213) and was not related to the severity of the disease [Tables 4 and 5].

**Table 4:** Laboratory test results

	Initially active AAV (A0)	AAV remission(A6)	P *	Control group	P**
**ANCA** (IU/ml)	51,0(8,1;123,0)	4,5(2,0;23,0)	**< 0,001**	0,2(0,1;0,2)	**< 0,001**
**Leukocytosis** (x10\cr^9/l)	9,6(8,64;12,62)	9,27(7,33;11,12)	0,262	6,41(5,24;7,96)	**< 0,001**
**Hemoglobin** (g/dl)	10,3(8,5;11,6)	11,3(11,0;12,6)	**0,002**	13,2(11,0;14,3)	**< 0,001**
**Platelets** (x10\cr^9/l)	269,0(221,0;321,0)	236,0(179,0;279,0)	**0,024**	239,0(188,0;285,0)	0,213
**ESR** (mm/1 h)	56,0(26,0;98,0)	17,0(13,0;32,0)	**< 0,001**	26,0(7,0;36,0)	**< 0,001**
**CRP** (mg/dl)	2,0(0,4;6,8)	0,2(0,1;0,4)	**< 0,001**	0,3(0,1;0,5)	**< 0,001**
**Procalcitonin** (ng/ml)	0,17(0,05;0,7)	0,05(0,03;0,14)	**< 0,001**	0,08(0,04;0,26)	0,094
**Albumin** (g/dl)	3,6(3,1;4,2)	4,3(4,1;4,5)	**< 0,001**	4,4(4,2;4,8)	**< 0,001**
**Fibrinogen** (mg/dl)	443,0(341,0;608,0)	379,0(281,0;465,0)	**0,011**	439,0(351,0;524,0)	0,208
**D-dimer** (µg/ml)	2,19(0,77;7,84)	0,79(0,4;2,06)	**0,004**	0,57(0,28;1,08)	**< 0,001**
**C3** (mg/dl)	114,0(100,0;1332,0)	106,0(97,0;127,0)	0,414	116,0(101,0;127,0)	0,876
**C4** (mg/dl)	23,0(18,0;27,0)	27,0(23,0;30,0)	**0,011**	25,0(22,0;29,0)	0,125

p* comparison of patients with active vasculitis and in remission, p** comparison of patients with active vasculitis and control group

**Table 5:** Comparison of test results depending on the severity of AAV

Disease form	ANCA(IU/ml)	WBC(x10,0\cr^9/l)	Hb(g/dl)	PLT(x10,0\cr^9/l)	ESR(mm/1 h)	CRP(mg/dl)
localized and early systemic *n* = 6	89,0(22,2;161,5)	11,49(8,75;14,6)	11,7(10,2;12,0)	240,5(153,7;328)	23,0(14,0;37,2)	0,25(0,1;1,75)
generalized *n* = 10	54,0(18,9;97,7)	8,9(7,98;11,3)	10,9(8,5;12,5)	262,5(232;451,7)	43,5(24,7;85,5)	1,4(0,35;3,9)
severe *n* = 12	53,0(5,92;171,5)	10,3(8,92;15,6)	8,65(7,7;10,1)	285,0(186;315,7)	85,0(57;111)	6,9(2,17;18)
P *	0,661	0,285	**0,009**	0,577	**0,007**	**0,006**
P**	0,685	0,639	**0,003**	0,685	**0,002**	**0,001**
**Disease form**	**PCT**(ng/ml)	**Albumin**(g/dl)	**Fibrinogen**(mg/dl)	**D-dimer**(µg/ml)	**C3**(mg/dl)	**C4** (mg/dl)
localized and early systemic *n* = 6	0,04(0,027;0,11)	4,3(3,9;4,5)	324(255;436)	1,08(0,49;4,04)	113,5(100;127)	28(23,5;29)
generalized *n* = 10	0,07(0,05;0,27)	3,65(3,15;4,37)	426(341;460)	0,77(0,48;2,39)	113,5(104;135)	22(17,7;24)
severe *n* = 12	0,91(0,4;2,94)	3,2(2,95;3,7)	590(485;716)	8,5(2,83;17,3)	124,0(98;134)	22(18;30)
P *	**0,000**	**0,043**	**0,009**	**0,001**	0,966	0,292
P**	**0,000**	**0,013**	**0,001**	**0,001**	0,875	0,333

Values are median (IQR)

P * in the Kruskal-Wallis test

P** in the Jonckheere-Terpstra test for the trend

The results of the study showed a strong relationship o the increased concentration of d-dimer and the activity and severity of systemic vasculitis. Prior to the initiation of remission-inducing treatment, the mean concentration of fibrin degradation products was as high as 6.59 µg/ml (median 2.19 µg/ml) and was significantly higher than in the control group (*p* < 0.001). Additionally, the result exceeded the reference value in 91 % of patients (no patient was clinically diagnosed with overt venous thrombosis). After 6 months of immunosuppressive treatment, the mean concentration of d-dimer decreased and returned to normal in the majority of patients (*p* = 0.004). [Tables 4 and 5]. Patients with active vasculitis were characterized by a lower concentration of hemoglobin and albumin in the peripheral blood compared to the control group and the evaluation performed in remission. These parameters also strongly correlated with the severity of the disease (*p* = 0.003, *p* = 0.013). Components of the C3 and C4 complement systems did not change significantly in the studied groups.

At the time of diagnosis of active AAV, the ANCA concentration was significantly increased, the mean value was 68.16 IU/ml, and the median was 51.0 IU/ml. The administered treatment decreased ANCA in the majority of patients, but the mean value was as high as 25.68 IU/ml (*p* < 0.001) [Tables 4 and 5]. Decrease of ANCA below the cut-off point for positive result was found in only 7 of 23 patients, while in 10 patients the ANCA concentration was only partially reduced. In the studied population, in one female patient, ANCA did not decrease and remained at a high level of > 177 IU/ml in complete clinical remission, in another woman the concentration of antibodies increased from 51 IU/ml to 78 IU/ml. In the remaining 4 patients, despite the low initial ANCA concentrations of 2.2–5.2 IU/ml, high clinical activity of the disease was observed, three patients were diagnosed with severe disease, and one with generalized disease. ANCA antibodies showed no correlation with the severity of GPA/MPA (*p* = 0.68) [Table 5].

## Discussion

The study analyzed the results of laboratory tests at different stages of AAV disease and treatment. The course of the disease is very variable, the assessment of the patient is based on experience and extensive differential diagnosis. The clinical activity scales BVAS v3 and BVAS/WG have a documented value for the assessment of the severity of the disease, but the classification of symptoms depends on the experience of the doctor [6].

The study population was characterized by high disease activity, more than 78 % of patients were diagnosed with the generalized and severe form. Renal involvement was confirmed in 75 % of patients, with initially high creatinine levels, which in most patients did not return to normal despite the treatment. The studies by Houben et al. and Hanaoka et al. showed that the increased concentration of creatinine and the decrease in GFR below 60 ml/min/1.73 m² at the time of diagnosis are late indicators of AAV activity in the kidneys, which is consistent with the presented work. Despite treatment, a significant proportion of patients remain in the advanced stage of chronic kidney disease, which significantly worsens the long-term prognosis [7, 8].

The study showed a strong correlation between the presence of microscopic hematuria and the activity of the disease in the kidneys. After achieving remission, erythrocyturia resolved in almost all patients. Monitoring of the urine sediment for the presence of red blood cells is of great importance for the assessment of inflammation within the glomeruli, and its resolution confirms remission, which has also been demonstrated in other studies [7–10]. The disappearance of proteinuria is likely to indicate remission of inflammation as well. Persistent proteinuria may be difficult to interpret, as it may result from irreversible changes in the kidneys or from the imposition of comorbidities such as hypertension or diabetes.

Patients with active vasculitis, particularly in the generalized and severe form, are characterized by a high intensity of inflammation. Secondary to inflammation, AAV patients had lower concentrations of hemoglobin and albumin in peripheral blood compared to the control group and to the evaluation performed in remission. These parameters strongly correlated with the severity of the disease (*p* = 0.003, *p* = 0.013). On the other hand, peripheral blood leukocytosis had a low value in AAV monitoring, which was also observed in the reports of other researchers [11]. However, studies demonstrated a high correlation of the increased concentration of CRP and the ESR value with AAV activity, as well as with the severity of the disease. After effective treatment, CRP concentration returned to normal in all patients, and ESR decreased significantly. Similar observations were made in numerous clinical trials, although the non-specificity of these parameters and the inability to differentiate from infectious complications were emphasized [12–14]. The study by Kalsch et al. also showed that an increase in CRP and an acceleration of OB are observed only in the presence of clinical symptoms of AAV activity and are not useful in predicting an impending exacerbation [15]. These parameters are also of low value in the assessment of localized and early systemic AAV, where the results may remain within the normal range despite the disease process.

The most important cause of death in the active phase of AAV are infectious complications, and the increase in inflammatory markers (leukocytosis, ESR, CRP) is equally typical for vasculitis and severe infection [16]. Repeated measurements of procalcitonin concentrations proved to be very helpful in the differentiation of bacterial infection, and their effectiveness in diagnostics has been demonstrated in many clinical studies [17, 18].

In the presented study, procalcitonin concentration was significantly higher in active vasculitis than in remission. The highest values were found in patients with severe AAV, who developed respiratory failure, alveolar bleeding, the need for mechanical ventilation or NIV assisted respiration. In all these patients broad-spectrum antibiotic therapy was used. Two patients from this population eventually died from septic shock.

The correct procalcitonin concentration of < 0.5 ng/ml is likely to exclude significant bacterial infection. In patients with AAV during the period of disease activity, results are often observed in the so-called gray zone (0.5-1.0 ng/ml), despite the absence of infectious symptoms, which may be due to a generalized inflammatory reaction. In the presented study, similarly to other clinical trials, procalcitonin concentration above 1.0 ng/ml was strongly associated with infectious complications, severe lower respiratory tract involvement and an increased risk of death [17, 18].

Numerous studies have shown an increased risk of venous thrombosis in the course of active AAV [19, 20]. The relationship of the markers stimulating the coagulation system and fibrinolysis with the activity of systemic vasculitis is less well documented [21]. The platelet counts in the majority of patients with active GPA/MPA (82.14 %) did not exceed the normal values, and the mean and median concentrations were low compared to the observations of other researchers [21]. In the presented work and in numerous other studies, an increased concentration of fibrinogen was observed in severe active vasculitis [21, 22]. The concentration of d-dimer proved to be a strong indicator of inflammation activity and a good marker of remission in other clinical studies as well [22, 23]. In the present work, a particularly high concentration of d-dimer was observed in the severe form of AAV (13.25 µg/ml), while in the generalized, early systemic and localized form it was significantly lower and did not differ significantly within these subgroups (2.08–1.84 µg/ml). Despite the increased risk of /hemorrhage in this population, increased vigilance and appropriate prophylaxis of thromboembolism are necessary due to the above observations.

The complement system has long not been associated with small-vessel vasculitis connected with the presence of ANCA. A significant role of alternative activation in the pathogenesis of this disease has been demonstrated only in recent years, [24–26]. The studies by Augusto et al. and Crnogorac et al. showed a worse prognosis of patients with regard to survival and renal function in the group with decreased component values of the C3 complement system,. However, no such relationship was found for the C4 component [26]. In the presented study, the concentrations of C3 and C4 complement components did not differ significantly depending on the activity of systemic vasculitis.

ANCA antibodies testing is crucial in the diagnosis of AAV. However, in monitoring disease activity, the interpretation of the results arouses controversy [5, 27–30].

The presented study shows that in the population with newly diagnosed AAV and in exacerbation, the concentration of ANCA antibodies is significantly higher than in remission. This is confirmed by the strong relationship between the amount of ANCA and AAV activity. As in the reports of other researchers, it has been shown that a significant decrease of ANCA concentration in the serum correlates well with the achievement of clinical remission [31–34]. It was demonstrated, however, that in the majority of patients in remission, ANCA antibodies were not completely eliminated, and despite the decrease in concentration, they still remained at a significant level. The present study shows that ANCA antibodies were still detected in the serum in 52 % of patients after 6 months of treatment despite the absence of disease activity (BVAS v3, BVAS/WG = 0 pts). We also observe patients whose ANCA concentration does not decrease or even increases despite effective treatment and the lack of clinical symptoms. In our study, ANCA antibodies also showed no correlation with AAV severity. This means that with high specificity for vasculitis, ANCA antibody measurements have limited sensitivity in disease monitoring, and any change in concentration should only be a guideline that requires interpretation in a clinical context [34]. The strengths of the study is a relatively numerous group of patients diagnosed and treated in one center. Moreover a large proportion of included patients who presented severe vasculitis with renal failure, pulmonary involvement and alveolar bleeding is also the advantage of the study. The analysis of frequently used inflammatory markers is extremely important in these patients to show that slight elevation of these markers is associated with disease flare and cannot delay the proper treatment. The main limitation of the study is it’s retrospective character.

In summary, the diagnosis of vasculitis is not easy, requires extensive clinical experience and access to immunological and histopathological tests. Appropriate treatment, both inductive and supportive, is even more challenging. The absence of markers unequivocally correlating with disease activity and differentiating from other clinical conditions makes management difficult. We expect that the results of the presented study will serve for a better interpretation of the available laboratory tests.

## Conclusions

Routinely performed laboratory tests such as CRP, ESR, fibrinogen, d-dimer, albumin and hemoglobin in the peripheral blood show a strong correlation with the clinical activity of AAV and at the same time well identify patients with severe disease.

Procalcitonin was slightly increased in patients with active AAV without infection. Its high values correlated with severe involvement of the respiratory system and showed a strong relationship with the occurrence of infectious complications. PCT monitoring may/can improve the safety of immunosuppressive therapy.

Repeated determinations of ANCA antibodies are useful in assessing AAV, although the expected relationship between the amount of antibodies and disease activity is not found in many patients. Therefore, the results should always be interpreted in the context of the occurrence of clinical symptoms.

The presented study showed that patients with high creatinine concentrations in the course of AAV remain in the advanced stage of chronic kidney disease, which significantly worsens the long-term prognosis. Microscopic hematuria was found to be an early and sensitive marker of disease activity, and its resolution indicated remission of glomerular inflammation.

## Data Availability

The datasets analyzed during the current study are available from the first author on reasonable request.

## Abbreviations

ACR: American College of Rheumatology
AAV: ANCA associated vasculitis
BVAS: Birmingham Vasculitis Activity Score
BVAS/WG: Birmingham Vasculitis Activity Score for Wegener`s Granulomatosis
CHCC: Chapel Hill Consensus Conference
CRP: C-reactive protein
EGPA: eosinophilic granulomatosis with polyangiitis
ESR: erythrocyte sedimentation rate
GCS: glucocorticosteroids
GFR: glomerular filtration rate
GPA: granulomatosis with polyangiitis
HD: hemodialysis
MPA: microscopic polyangiitis
PCT: procalcitonin
RLV: renal limited-AAV
VDI: Vasculitis Damage Index

## References

[1] Leavitt RY, Fauci AS, Bloch DA, Michel BA, Hunder GG, Arend WP (1990). The American College of Rheumatology 1990 criteria for the classification of Wegener’s granulomatosis. Arthritis Rheum..

[2] Jennette JC, Falk RJ, Bacon PA, Basu N, Cid MC, Ferrario F (2013). 2012 Revised International Chapel Hill Consensus Conference Nomenclature of Vasculitides. Arthritis Rheum..

[3] Yates M, Watts RA, Bajema IM, Cid MC, Crestani B, Hauser T (2016). EULAR/ERA-EDTA recommendations for the management of ANCA-associated vasculitis. Ann Rheum Dis.

[4] Wiatr E, Gawryluk D (2013). Pierwotne systemowe zapalenia naczyń związane z przeciwciałami przeciw-cytoplazmatycznymi (ANCA) — rekomendacje diagnostyczne i lecznicze: Pneumo¬nol. Alergol. Pol.

[5] Stegeman CA (2002). Anti-neutrophil cytoplasmic antibody (ANCA) levels directed against proteinase-3 and myeloperoxidase are helpful in predicting disease relapse in ANCA-associated small-vessel vasculitis. Nephrol Dial Transplant.

[6] Suppiah R, Mukhtyar C, Flossmann O, Alberici F, Baslund B, Batra R (2011). A cross-sectional study of the Birmingham Vasculitis Activity Score version 3 in systemic vasculitis. Rheumatology.

[7] Houben E, van der Heijden JW, van Dam B, Bax WA, Voskuyl AE, Penne EL (2017). Screening for renal involvement in ANCA-associated vasculitis: room for improvement?. Neth J Med.

[8] Hanaoka H, Ota Y, Takeuchi T, Kuwana M (2016). Poor renal outcomes in patients with anti-neutrophil cytoplasmic antibody-associated crescentic glomerulonephritis and normal renal function at diagnosis. Clin Rheumatol..

[9] Geetha D, Seo P, Ellis C, Kuperman M, Levine SM (2012). Persistent or new onset microscopic hematuria in patients with small vessel vasculitis in remission: findings on renal biopsy. J Rheumatol..

[10] Lv L, Chang DY, Li ZY, Chen M, Hu Z, Zhao MH (2017). Persistent hematuria in patients with antineutrophil cytoplasmic antibody-associated vasculitis during clinical remission: chronic glomerular lesion or low-grade active renal vasculitis?. BMC Nephrol.

[11] Cartin-Ceba R, Peikert T, Specks U (2010). Pathogenesis of ANCA-Associated Vasculitis. Rheum Dis Clin North Am.

[12] Monach PA, Warner RL, Tomasson G, Specks U, Stone JH, Ding L (2013). Serum proteins reflecting inflammation, injury and repair as biomarkers of disease activity in ANCA-associated vasculitis. Ann Rheum Dis.

[13] Hind CR, Winearls CG, Lockwood CM, Rees AJ, Pepys MB (1984). Objective monitoring of activity in Wegener’s granulomatosis by measurement of serum C-reactive protein concentration. Clin Nephrol.

[14] Csernok E, Bossuyt X (2018). Investigations in systemic vasculitis. The role of the laboratory. Best Pract Res Clin Rheumatol..

[15] Kälsch AI, Csernok E, Münch D, Birck R, Yard BA, Gross W (2010). Use of highly sensitive C-reactive protein for followup of Wegener’s granulomatosis. J Rheumatol..

[16] Yang L, Xie H, Liu Z, Chen Y, Wang J, Zhang H (2018). Risk factors for infectious complications of ANCA-associated vasculitis: a cohort study. BMC Nephrol.

[17] Eberhard OK, Haubitz M, Brunkhorst FM, Kliem V, Koch KM, Brunkhorst R (1997). Usefulness of procalcitonin for differentiation between activity of systemic autoimmune disease (systemic lupus erythematosus/systemic antineutrophil cytoplasmic antibody-associated vasculitis) and invasive bacterial infection. Arthritis Rheum..

[18] Moosig F, Csernok E, Reinhold-Keller E, Schmitt W, Gross WL (1998). Elevated procalcitonin levels in active Wegener’s granulomatosis. J Rheumatol..

[19] Springer J, Villa-Forte A (2013). Thrombosis in vasculitis. Curr Opin Rheumatol..

[20] Allenbach Y, Seror R, Pagnoux C, Teixeira L, Guilpain P, Guillevin L, French Vasculitis Study Group (2009). High frequency of venous thromboembolic events in Churg-Strauss syndrome, Wegener’s granulomatosis and microscopic polyangiitis but not polyarteritis nodosa: a systematic retrospective study on 1130 patients. Ann Rheum Dis..

[21] Willeke P, Kümpers P, Schlüter B, Limani A, Becker H, Schotte H (2015). Platelet counts as a biomarker in ANCA-associated vasculitis. Scand J Rheumatol.

[22] Ma TT, Huang YM, Wang C, Zhao MH, Chen M (2014). Coagulation and fibrinolysis index profile in patients with ANCA-associated vasculitis. PLoS One..

[23] Hergesell O, Andrassy K, Nawroth P (1996). Elevated levels of markers of endothelial cell damage and markers of activated coagulation in patients with systemic necrotizing vasculitis. Thromb Haemost..

[24] Yuan J, Gou SJ, Huang J, Hao J, Chen M, Zhao MH (2012). C5a and its receptors in human anti-neutrophil cytoplasmic antibody (ANCA)-associated vasculitis. Arthritis Res Ther..

[25] Chen M, Daha MR, Kallenberg CG (2010). The complement system in systemic autoimmune disease. J Autoimmun.

[26] Augusto JF, Langs V, Demiselle J, Lavigne C, Brilland B, Duveau A (2016). Low Serum Complement C3 Levels at Diagnosis of Renal ANCA-Associated Vasculitis Is Associated with Poor Prognosis. PLoS One..

[27] Hagen EC, Daha MR, Hermans J, Andrassy K, Csernok E, Gaskin G (1998). Diagnostic value of standardized assays for anti-neutrophil cytoplasmic antibodies in idiopathic systemic vasculitis. ECuBCR Project for ANCA Assay Standardization. Kidney Int.

[28] Girard T, Mahr A, Noel L-H, Cordier J-F, Lesavvre P, Andre M-H, Guillevin L (2001). Are antineutrophil cytoplasmic antibodies a marker of relapse in Wegener’s granulomatosis? A prospective study. Rheumatology (Oxford).

[29] Boomsma MM, Stegeman CA, van der Leij MJ, Oost W, Hermans J, Kallenberg CG (2000). Prediction of relapses in Wegener’s granulomatosis by measurement of antineutrophil cytoplasmic antibody levels: a prospective study. Arthritis Rheum.

[30] Finkielman JD, Merkel PA, Schroeder D, Hoffman GS, Spiera R, St Clair EW, WGET Research Group (2007). Antiproteinase 3 antineutrophil cytoplasmic antibodies and disease activity in Wegener granulomatosis. Ann Intern Med.

[31] Specks U, Wheatley CL, McDonald TJ, Rohrbach MS, DeRemee RA (1989). Anticytoplasmic autoantibodies in the diagnosis and follow-up of Wegener’s granulomatosis. Mayo Clin Proc..

[32] Tervaert JW, van der Woude FJ, Fauci AS, Ambrus JL, Velosa J, Keane WF (1989). Association between active Wegener’s granulomatosis and anticytoplasmic antibodies. Arch Intern Med.

[33] Terrier B, Saadoun D, Sène D, Ghillani P, Amoura Z, Deray G (2009). Antimyeloperoxidase antibodies are a useful marker of disease activity in antineutrophil cytoplasmic antibody-associated vasculitides. Ann Rheum Dis..

[34] Jennette JC, Nachman PH (2017). ANCA Glomerulonephritis and Vasculitis. Clin J Am Soc Nephrol.

